# The Use of Resuscitative Endovascular Balloon Occlusion of the Aorta in a Case of Suspected Septic Distributive Shock: A Case Report

**DOI:** 10.1016/j.acepjo.2025.100088

**Published:** 2025-03-14

**Authors:** Peter Hilbert-Carius, Astrit Heiser, Hermann Wrigge, Pia Hölbing, Patrick Schröter, Philipp Kobbe, Axel Großstück

**Affiliations:** 1 Department of Anaesthesiology, Intensive Care, Emergency Medicine and Pain Therapy, Trauma Centre Bergmannstrost Halle (Saale), Halle (Saale), Germany; 2 Medical Faculty, Martin-Luther-University Halle-Wittenberg, Halle (Saale), Halle (Saale), Germany; 3 Department of Plastic and Hand Surgery, Burn Unit, Trauma Centre Bergmannstrost Halle (Saale), Halle (Saale), Germany; 4 Department of Trauma and Reconstructive Surgery, Trauma Centre Bergmannstrost Halle (Saale), Halle (Saale), Germany; 5 Department of Trauma and Reconstructive Surgery, University Hospital Halle (Saale), Halle (Saale), Germany

**Keywords:** resuscitative endovascular balloon occlusion of the aorta (REBOA), CPR, distributive shock, osteomyelitis, vasopressor, aortic occlusion

## Abstract

Resuscitative endovascular balloon occlusion of the aorta (REBOA) is mainly used in patients with major noncompressible torso hemorrhage and more recently as an adjunct in cardiopulmonary resuscitation to improve coronary and cerebral perfusion pressure during chest compressions. The use of partial REBOA as a resuscitative adjunct in distributive shock like septic or anaphylactic shock is not a current indication of its use. Nevertheless, the use of partial REBOA for the early incidence of profound distributive shock with the need for massive vasopressor support can be an option and a bridge to stabilize the patient until further treatment can be administered. We presented a case of a patient with intraoperative profound septic shock due to the release of inflammatory mediators from purulent osteomyelitis during marrow canal reaming. Due to massive vasodilatation refractory to vasopressor and fluid resuscitation, the patient needed a short period of mechanical chest compression. After REBOA placement in zone I with partial REBOA, the patient became stable, and the vasopressors could be decreased. Within the next hour, due to the use of volume resuscitation and antibiotics, the patient became more and more stable, and REBOA could slowly be deflated. With deflated REBOA still in place, the patient remained stable in the intensive care unit and infection remediation through amputation of both lower legs could be carried out on the same day. The patient was discharged home without a neurologic deficit 6 weeks later. In a situation in which fluid resuscitation and the use of vasopressor cannot stabilize the patient in distributive shock, partial REBOA might be an option to restore central perfusion until further measures can take effect. In the described case, partial REBOA proved to be effective and was able to bridge the time until definitive care was effectively undertaken.

## 1 Introduction

Resuscitative endovascular balloon occlusion of the aorta (REBOA) is a minimally invasive procedure being increasingly utilized to prevent patients with noncompressible torso hemorrhage from exsanguination. It is used as a bridge to surgical bleeding control to gain time during the management of hemorrhagic shock as part of the endovascular resuscitation and trauma management (EVTM) concept.^1^ More recently, REBOA has been utilized in cardiopulmonary resuscitation (CPR) to increase coronary and cerebral perfusion pressure to achieve a return of spontaneous circulation and to improve neurologic outcomes.^2^, ^3^, ^4^, ^5^, ^6^, ^7^ Access to the common femoral artery (CFA) is essential to advance the REBOA catheter via a sheath into the desired landing zone of the aorta. Zone I (from the left subclavian artery to the celiac trunk) is the landing zone to stop intraabdominal bleeding and to increase coronary and cerebral perfusion pressure during CPR. Zone II (from the celiac trunk to the lowest renal artery) is called the nonocclusion zone. Zone III (from below the lowest renal artery to the aortic bifurcation) is the landing zone for pelvic bleeding, postpartum hemorrhage, or placental accreta bleeding.

On the other hand, patients with distributive shock, eg, due to sepsis or anaphylaxis, with the need for massive vasopressor support, have a similar problem as patients with hemorrhagic shock with massive volume requirements due to profound vasodilatation. The use of vasopressors and volume resuscitation is common in these patients. However, some patients are refractory to vasopressors and are at risk of cardiac arrest due to low diastolic pressure and consecutive low coronary perfusion. The use of REBOA in other etiologies of shock, such as distributive shock from sepsis, has not been investigated in humans. However, in cases refractory to vasopressor support and immediate risk of cardiac arrest, the use of REBOA has the theoretical potential of temporarily augmenting mean arterial pressure until definitive stabilization. We present a case of a patient with a suspected distributive shock that occurred intraoperatively, leading to peri-arrest. We observed that the use of partial REBOA was able to bridge the time until other measures to stabilize the patient could be administered.

Ethical approval was not necessary, as this was not a study or experimental work, and REBOA is a standard procedure in our hospital.

## 2 Case

A 30-year-old Ukraine soldier was brought to our level-1 trauma and burn center 20 days after suffering an explosion trauma with the following injuries:

- Complex comminuted fractures of both lower legs after shrapnel injury
- Injuries due to blast trauma
- Mid-dermal to deep dermal-thickness burns of a total of approximately 20% of body surface area, affecting the head, left ear, both hands, partially both legs and feet
- Inhalation trauma

The initial treatment of the patient took place in Ukraine, where the fractured legs were stabilized with external fixation, and daily surgical wound treatment with antiseptic solutions for the burned skin was performed. Antimicrobial treatment was initiated in Ukraine, and multiple multiresistant germs (4 multiresistant Gram-negative bacteria [MRGN]: *Pseudomonas aeruginosa*, 4 MRGN *Klebsiella pneumoniae*, methicillin-resistant *Staphylococcus aureus* [MRSA]) and osteomyelitis of both injured legs were detected on admission to our center.

In our center, the initial course of treatment of the burns and corresponding split-thickness skin grafts was completed without complications before surgical treatment of the infected and fractured lower legs.

About 1.5 months after the initial injury, the 2 injured lower extremities were operated on, and the medullary cavity was carefully reaming and irrigated to treat the known osteomyelitis. Just before the end of the operation, a massive circulatory collapse occurred without major blood loss. Before the operation, the patient was supplied with a central venous catheter and an arterial line and was in stable conditions without any vasopressor support and spontaneous breathing. Despite the immediate use of vasopressors (initially with push-doses of theodrenalin-cafedrin -Akrinor- and then adrenaline and noradrenaline continuously each with 0.1 μg/kg/min), forced administration of balanced fluids and an increase in inspiratory oxygen to 100%, the progression of circulatory failure with a concomitant decrease in expiratory CO_2_ from 38 mm Hg to 20 mm Hg could not be prevented. The ventilator setting (pressure-controlled ventilation—PSV, positive end-expiratory pressure—PEEP 7 mbar, positive inspiratory pressure—PIP 20 mbar, respiratory rate—RR 14/min, minute volume—MV 7.5 L/min, fraction of inspiratory oxygen—FiO_2_ 0.35) were not changed throughout the operation. The subject had a heart rhythm but only generated extremely low blood pressure (BP) of 35/20 mm Hg. Therefore, CPR was started, initially with manual chest compressions and then with an automated chest compression device (ACCD, Lucas III, Lund University, Sweden). After an initial push-dose of 1 mg of adrenaline, the administration of adrenaline (0.5 μg/kg/min) and noradrenaline (0.3 μg/kg/min) was continued. As shown in the Figure, chest compressions, and adrenaline did not increase the recorded BP.

**Figure:**
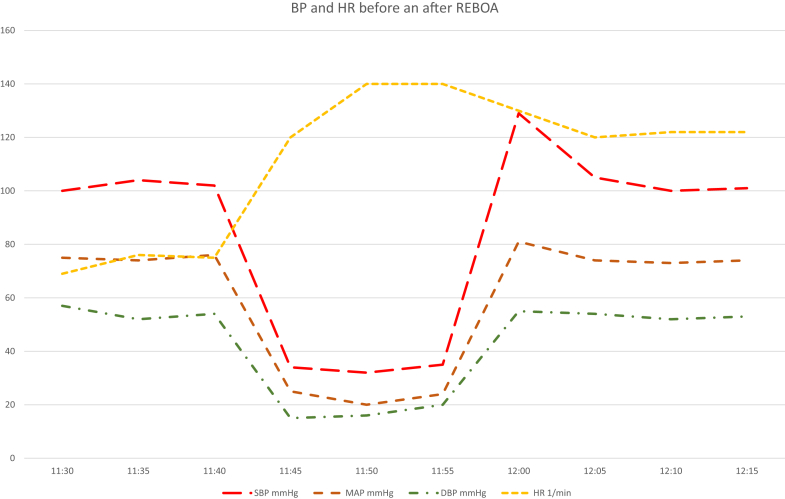
Time course of blood pressure and heart rate before hemodynamic collapse (11:30-11:40 AM), during CPR (11:42-11:56 AM), and after REBOA (12:00-12:15 PM). Partial REBOA was initiated at approximately 12:02 PM. **†** start of manual chest compression; **‡** ACCD started; **∗** occlusion of the aorta; **∗∗** start of partial occlusion. ACDD, automated chest compression device; CPR, cardiopulmonary resuscitation; DBP, diastolic blood pressure; HR, heart rate; MAP, mean arterial pressure; REBOA, resuscitative endovascular balloon occlusion of the aorta; SBP, systolic blood pressure.

To improve coronary and cerebral perfusion, we decided to use a REBOA device. In our hospital, REBOA is regularly used in the trauma bay and within the scope of the RIBCAP-HEMS project (REBOA in bleeding and cardiac arrest in the prehospital care using helicopter emergency medical service) in a prehospital setting by consultant emergency physicians and anesthetists. A 7F sheath was inserted through the right CFA using ultrasound. Owing to the suspicion of a possible pulmonary embolism (PE), the patient received 5000 IU heparin and 10 mg alteplase intravenously following the collection of blood samples after sheath implantation. Through the 7F sheath, an ER-REBOA PLUS catheter (Prytime Medical) was inserted into zone I above the diaphragm. The ACCD was stopped, and immediately after total occlusion of the aorta, the BP above the balloon increased to 129/55 mm Hg, allowing for a substantial reduction in catecholamines to 0.1 μg/kg/min of adrenaline and 0.2 μg/kg/min noradrenaline. To limit the amount of ischemia, we decided to use partial REBOA. The BP above the balloon dropped to approximately 100/50 mm Hg, and the BP below the balloon, as measured through the side port of the sheath, was approximately 55/30 mm Hg. This meant that blood still flowed to the organs distal to the partial occlusion of the aorta. After stabilization due to REBOA, the electrocardiogram did not show any signs of ST elevation or disturbances of repolarization. Unfortunately, an echocardiogram was not performed in the operating room.

After temporary wound closure and ongoing fluid resuscitation, the patient was transferred to the computed tomography (CT) scanner to scan for PE or intraabdominal causes of circulatory collapse. No PE or intraabdominal problems were detected as reasons for the circulatory problems. Partial REBOA was continued during the diagnostic procedure, then the patient was transferred to the intensive care unit (ICU), and fluid resuscitation was continued. Vasopressor support was reduced with further fluid therapy and escalation of antimicrobial therapy (ceftazidime/avibactam, linezolid, and aztreonam/avibactam), and partial occlusion of the aorta was completely stopped within 50 minutes after the initiation of REBOA. During the time from the beginning of vascular collapse to the total deflation of the REBOA balloon, the patient received 3000 mL of balanced crystalloids.

Based on the unremarkable CT scan results, the laboratory results now available (see Table), and the usual workup for severe shock, fulminant distributive septic shock therefore appeared to be the most plausible cause of massive circulatory failure.

**Table:** Laboratory results before the operation, after the operation, and on the first postoperative day.

Parameters	Preoperative	Postoperative	First postoperative day
WBC Gpt/L	5.7	43.3	21.2
Platelets Gpt/L	385	715	412
CRP mg/L	16	69	96
PCT ng/mL	0.04	73	43.1
D-Dimer mg/L	0.34	5.31	n.m.
Troponin T (high sensitive) ng/L	n.m.	5.4	n.m.
Lactate mmol/L	1.3	9.1	1.7
Base excess	2.3	−13.3	2.8
Potassium mmol/L	4.7	4.3	5.0

CRP, C-reactive protein; Gpt/L, 10^9^/L; n.m., not measured; PCT, procalcitonin; WBC, white blood cell.

Owing to massive septic shock with pronounced osteomyelitis of both lower extremities as the probable cause of sepsis, the decision was made to disarticulate both lower legs for focal sanitation on the same day. The inserted REBOA catheter remained in situ for the procedure to be able to react in the event of massive blood loss or renewed septic circulatory failure. However, it was not necessary to block the catheter again so that it could be removed postoperatively. After this procedure, the patient continued to stabilize. The 7F sheath of the right CFA was removed 4 hours after surgery in the ICU without complications. During the further course of treatment, a revision operation was necessary, but no further septic episodes occurred. Two weeks later, the patient was transferred from the ICU to the normal ward, and 4 weeks later, he was discharged to his home country without any neurologic impairment. During the ICU, no impairment in kidney function occurred. The patient had normal urine output and daily estimated glomerular filtration rates (eGFRs) using the Chronic Kidney Disease Epidemiology Collaboration (CKD-EPI) formula within normal ranges.

## 3 Discussion

REBOA, the original therapeutic method for traumatic noncompressible torso hemorrhage (NCTH), was developed in the 1950s and is now used for significantly more indications and even in prehospital settings.^2^, ^3^, ^4^, ^5^, ^6^, ^7^ To our knowledge, the use of REBOA in distributive septic shock has not yet been described in the literature. In septic shock, mediator release can lead to endothelial injury, tissue hypoperfusion, disseminated intravascular coagulation, and refractory shock.^8^ Vascular paralysis is correlated with prognosis in septic shock patients, and nonsurvivors have significantly more pronounced vascular paralysis.^9^ In such cases, the diastolic BP is extremely low and not sufficient to meet the requirements of the myocardium. Ultimately, in addition to distributive shock, this leads to cardiogenic shock due to insufficient myocardial perfusion and thus may require resuscitation. As shown in the Figure, conventional resuscitation with chest compressions and intravenous adrenaline did not increase the recorded BP and therefore was unlikely to increase coronary and cerebral perfusion, 2 important predictors of successful resuscitation. There is evidence from animal studies that REBOA during CPR increases BP and coronary and cerebral perfusion, yet human data are relatively sparse and mainly based on case reports and small case series.^3^^,^^10^

One possible differential diagnosis besides the initially suspected PE is fat emboli syndrome.^11^ Based on our experience, the syndrome is very rare, even in patients with medullary cavity reaming. On the other hand, the laboratory results did show a clear picture regarding sepsis, thus excluding fat emboli syndrome as a cause of the deterioration of the patient.

The use of REBOA in distributive shock is a completely new field but does make sense in light of the abovementioned pathophysiologic fundamentals. In a situation in which fluid resuscitation and the use of vasopressors cannot stabilize the patient, partial REBOA might be an option to restore central perfusion until further measures can take effect, as described in our case. Two animal studies have shown that endovascular perfusion augmentation for critical care (EPACC), meaning that an endovascular balloon catheter is used for hemodynamic support in shock states, provides improved renal flow with improvement in terminal creatinine levels compared with standard critical care (SCC) with stabilized proximal hemodynamic and a decreased vasopressor dose.^12^^,^^13^ Different studies have shown that during the EPACC, there is no statistically significant decrease in blood flow at the end-organ level and that animals in the EPACC group have greater BP, require less intravenous fluids, and that there are no differences in renal cellular damage on histology compared with animals in the SCC group.^12^, ^13^, ^14^

EPAC is provided by an automated care platform consisting of a central processing unit (CPU) that receives physiologic data from a data acquisition system. The CPU-transmitted instructions are based on predefined algorithms for 3 peripheral devices: an automated syringe pump controlling the endovascular balloon, a syringe pump titrating the administration of norepinephrine, and a peristaltic pump providing intravenous (IV) crystalloid boluses.^14^ In our case, we did the tasks of the automated care platform manually. We adjusted the balloon, guided the vasopressor, and performed fluid resuscitation according to the patient’s response.

The supported hemodynamics with increased coronary and cerebral perfusion pressure, along with the use of partial REBOA in distributive shock and the maintenance of blood flow at the end-organ level below the balloon, made the procedure a very interesting and meaningful way to address pronounced distributive shock. Catheters for partial REBOA are now available; these catheters would be suitable in such a situation as a true resuscitation adjunct.^15^^,^^16^

In conclusion, massive distributive shock can lead to total collapse of the cardiopulmonary system with a subsequent need for CPR. Partial REBOA may be a viable resuscitation adjunct in such cases to restore hemodynamics and increase coronary and cerebral perfusion pressure without reducing end-organ blood flow. Partial REBOA can bridge the time until other measures to stabilize the patient can be administered. Further animal and human clinical studies are needed to prove, or disprove, the potential beneficial effect of partial REBOA in distributive shock.

## Funding and Support

By *JACEP Open* policy, all authors are required to disclose any and all commercial, financial, and other relationships in any way related to the subject of this article as per ICMJE conflict of interest guidelines (see www.icmje.org). The authors have stated that no such relationships exist.

## Conflict of Interest

Peter Hilbert-Carius received study support (third-party funding) from DRF Luftrettung for his ongoing study “REBOA in bleeding and cardiac arrest in the prehospital care by helicopter emergency medical service.” He is a member of the German Society of Anaesthesiology and Intensive Care Medicine (Deutsche Gesellschaft für Anästhesiologie und Intensivmedizin, DGAI), the Association of Emergency Physicians in Saxony-Anhalt (Arbeitsgemeinschaft der in Sachsen-Anhalt tätigen otärzte, AGSAN), and the EVTM Society. All other authors declared that they have no known competing financial interests or personal relationships that could have appeared to influence the work reported in this paper.
